# TESTING THE MODERATING EFFECTS OF DEPRESSIVE SYMPTOMS ON A PHYSICAL ACTIVITY INTERVENTION

**DOI:** 10.1093/geroni/igac059.359

**Published:** 2022-12-20

**Authors:** Emily Smail, Christopher Kaufmann, Todd Manini

**Affiliations:** University of Florida, Gainesville, Florida, United States; University of Florida, Gainesville, Florida, United States; University of Florida, Gainesville, Florida, United States

## Abstract

Depressive symptoms affect the physical and cognitive health of approximately 20% of older adults. These symptoms have strong, bidirectional ties with physical activity levels and mobility disability. Physical activity has a positive impact on mood and depression and is highly recommended for symptom management across all ages. However, it’s unclear whether elevated depressive symptoms interfere with potential benefits that physical activity has on other health outcomes like mobility loss. The Lifestyle Interventions and Independence for Elders (LIFE) Study randomized over 1,500 older adults (aged 70+) to either a physical activity (intervention) or successful aging (control) program with an average of 2.2 years of follow-up. Our analysis used Cox proportional hazards models to evaluate whether elevated depressive symptoms (measured using the Center for Epidemiological Studies-Depression (CESD)-11 scale with a cutoff score of 16/22 points) moderated the relationship between intervention status and the primary outcome (incident major mobility disability, objectively measured as the ability to walk 400 meters). In a secondary analysis of 1,534 older adults (Mage = 78.8, 66.7% female), we confirmed significant main effects of both the physical activity intervention and elevated depressive symptoms on incident major mobility disability but found no evidence of moderation (interaction p-value=0.989). Results indicate that the benefits of the intervention were comparable between those with and without significant depressive symptoms at baseline, supporting the inclusion of individuals with depression in clinical trials. In addition to potential symptom relief, promoting physical activity in older adults with depression is important for protecting against mobility loss.

